# Corrigendum

**DOI:** 10.1002/ehf2.13551

**Published:** 2021-08-02

In the paper by Huttin *et al*.,^1^ the authors reported that there was a typo in *Figure* *3*. Indeed, titles of panels B and C were the same. The correct version is shown below.

**Figure 3:**
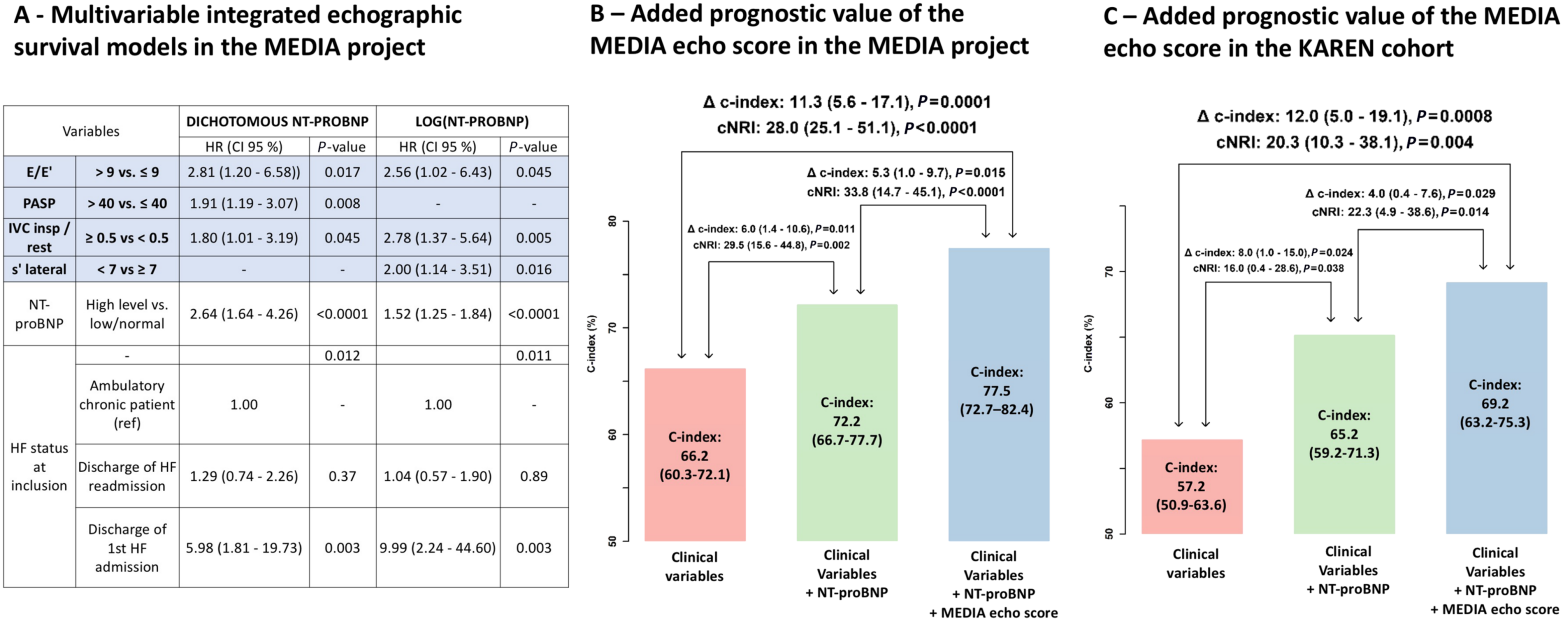
Multivariable integrated echographic models in the MEDIA project (panel A) and its added prognostic value in the MEDIA project (panel B) and the KaRen cohort (panel C). Panel A: Cox regression model, using subset of variables retained after backward selection (using missing‐indicator method) with NT‐proBNP as a dichotomous or linear variable; Panel B and C: Improvement in prognostic value for the primary end‐point on top of clinical model (including age, eGFR, AF and HF status), assessed by NRI and C‐index.

The authors apologize for this error.
